# Incidental Splenic Marginal Zone Lymphoma With Extreme Macrocytosis After Hydroxyurea Use: A Case Report

**DOI:** 10.7759/cureus.33462

**Published:** 2023-01-06

**Authors:** Kinza Sultan, Sarala Kal, Andrew Wasson, Farbod Farmand

**Affiliations:** 1 Internal Medicine, Arrowhead Regional Medical Center, Colton, USA

**Keywords:** large b-cell lymphoma, secondary malignancy, non-hodgkin lymphoma, splenic marginal zone lymphoma, hydroxyurea use

## Abstract

Splenic marginal zone lymphoma (SMZL) is a low-grade mature B-cell lymphoma that typically presents in the form of splenomegaly and lymphocytosis. The diagnosis is traditionally made through splenic histology, the presence of circulating villous lymphocytes, or bone marrow biopsy. Its treatment can be in the form of chemotherapy, such as rituximab, or active surveillance. This case presentation discusses a 76-year-old female with a long history of hydroxyurea use for an unknown reason presenting with atypical symptoms requiring bone marrow biopsy to diagnose SMZL. This unique case demonstrates the importance of further research and studies into atypical SMZL presentations and hydroxyurea’s potential in precipitating secondary malignancies.

## Introduction

Splenic marginal zone lymphoma (SMZL) is a low-grade, mature B-cell lymphoma that is categorized as non-Hodgkin lymphoma (NHL) and subclassified as a marginal zone lymphoma (MZL) representing approximately 0.9% of NHLs and 20% of MZLs [1]. The majority of SMZLs present with obvious splenomegaly due to localized encroachment and replacement of the splenic white pulp by aberrant memory B-cells. Dissemination of pathologic lymphocytes may involve the bone marrow and blood, but rarely includes peripheral lymph nodes and extra-nodal sites [2]. Most SMZL patients present with laboratory findings significant for anemia, thrombocytopenia, and lymphocytosis [3].

The World Health Organization (WHO) classified SMZL as a distinct pathology in 1991 [4]. Prior cases have been described, including splenic B-cell lymphomas with circulating villous lymphocytes (SLVL), believed to represent the same pathology as SMZL in different stages [5,6]. The median age of diagnosis is 65 years old with an age-adjusted incidence of 0.13/100,000 people per year [7]. SMZL is equally prevalent in both genders. Traditionally, the diagnosis was achieved via splenic histology and/or the presence of circulating villous lymphocytes. However, bone marrow biopsy has become an accepted substitution [8]. Survival rates have increased to >10 years, likely due to SMZL’s indolent course and the prevalence of rituximab as a first-line monotherapy [9]. Shortened survival is associated with diagnosis after 60 years old, Hispanic ethnicity, presence of B-cell symptoms, and treatment with non-rituximab chemotherapy [10].

Here, we present a unique case of a 76-year-old female who presented with fatigue and malaise and was found to have extreme macrocytosis. Further evaluation revealed an SMZL that transformed into diffuse large B-cell lymphoma.

This case report was carried out in accordance with the Institutional Review Boards for Human Research at Arrowhead Regional Medical Center in Colton, CA. The subject gave written informed consent and the respective Institutional Review Boards approved the protocol.

## Case presentation

A 76-year-old-female with a past medical history of heart failure with a preserved ejection fraction of 65% (HFpEF), a benign anterior fossa tumor with resection in May of 2019, unknown disorder causing thrombocytosis treated with hydroxyurea, and transient ischemic attack in 2006 presented with complaints of worsening malaise, weakness, hematochezia over several weeks, and chest tightness over several days. Home medications included Atenolol 50 mg PO qD, hydrochlorothiazide 25 mg PO qD, and hydroxyurea 500 mg PO TID. On physical exam, she appeared fatigued with poor respiratory effort, right-upper quadrant abdominal tenderness, pitting edema in bilateral lower extremities, and a sparse petechial rash on her bilateral lower extremities.

Upon arrival, vitals were unremarkable. Initial labs were significant for macrocytic anemia (hemoglobin of 7.8 g/dL, mean corpuscular volume (MCV) of 146 fL), and thrombocytopenia (platelet count of 15 x103/uL). Stool guaiac was positive. The basic metabolic panel was significant for hyponatremia of 126 mmol/L, CO_2_ of 17 mmol/L, elevated blood urea nitrogen of 38 mg/dL, and elevated brain-natriuretic peptide (BNP) of 1590 pg/mL. All other values, such as white blood cell count, absolute neutrophil count, glomerular filtration rate (GFR), creatinine, electrocardiogram (EKG), and cardiac enzymes, were within normal limits. Abdominal ultrasound revealed mild fatty liver changes with no hepatosplenomegaly.

She was admitted for management of heart failure exacerbation and possible gastrointestinal (GI) bleed. Cardiac workups, including monitoring for arrhythmia on telemetry, serial cardiac troponins, and ECGs, were all unremarkable. Transthoracic echo showed preserved ejection fraction with grade I diastolic dysfunction. Treatment with furosemide intravenously (IV) and fluid restriction <1.5 Liters/day improved both volume status and hyponatremia.

Gastroenterology was consulted due to concern for GI bleed given the reported hematochezia with positive hemoccult testing; esophagogastroduodenoscopy (EGD) and colonoscopy were deferred given thrombocytopenia. The patient was started on pantoprazole 40 mg IV BID empirically and did not have any recurrence of hematochezia.

Evaluation of the patient’s anemia included an iron panel that was consistent with anemia of inflammatory etiology with an elevated reticulocyte percentage of 12.4 and a reticulocyte index of 3.2. This was consistent with blood loss with appropriate bone marrow response. D-Dimer and LDH were elevated at 470 ng/mL and 800 U/L respectively, however, serology with normal bilirubin, negative DIC panel, and negative coombs testing made active hemolysis unlikely. Serum folate and cobalamin levels were within normal limits, making nutritional deficiency unlikely. Blood smear revealed few hyper-segmented nuclei and no schistocytes.

**Figure 1:**
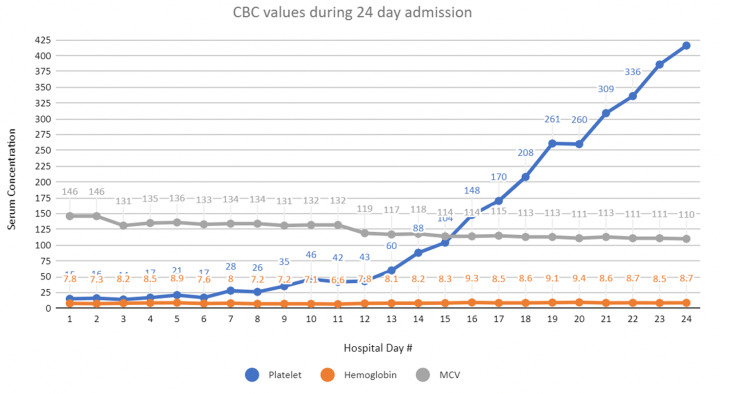
CBC values during the course of the hospital stay, including platelets, hemoglobin, and MCV CBC: complete blood count; MCV: mean corpuscular volume

Hydroxyurea was held, which improved cell counts as depicted in Figure 1. However, cytopenia persisted, and given that there was no other clear etiology to the patient's cytopenia, she underwent bone marrow biopsy, which revealed variably normocellular to hypocellular marrow for the patient’s age with cellularity of 10-20%, as well as diminished active trilineage hematopoiesis composed of maturing myeloid and erythroid series without any significant dyspoietic features. The bone marrow biopsy was significant for 10-20% of focal atypical cells suggestive of lymphoid differentiation. Flow cytometry showed expression of clonal CD20+ B-cells that co-express CD5/CD23 and surface Kappa light chain comprising 20% of all cells. Further analysis confirmed a low-grade, B-cell lymphoproliferative disorder with approximately 15% interstitial and intrasinusoidal involvement of cellular marrow. The most likely diagnosis was CD5-positive SMZL, especially given the intrasinusoidal involvement of cellular marrow.

**Figure 2:**
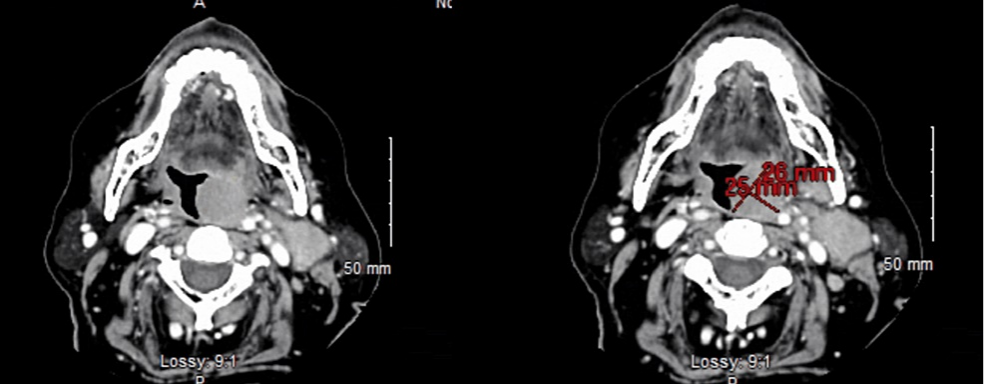
CT maxillofacial with IV contrast depicting a 25 mm x 26 mm oropharyngeal mass

During the hospital stay, the patient also complained of odynophagia and neck pain with anterior cervical lymphadenopathy. CT maxillofacial illustrated a 2.6 cm homogeneous lesion in the left oropharynx suspicious of a solid mass as seen in Figure 2. ENT was consulted and the mass was biopsied, with pathology significant for large B-cell lymphoma, concerning for large cell transformation. The patient was eventually transferred to a skilled rehabilitation facility and appropriate therapy was started in the outpatient setting by oncology.

## Discussion

The pathogenesis of SMZL involves acquired mutations and genomic instability in 75% of cases [11]. Unlike other lymphocytic cancers, which often involve the inactivation of tumor suppressor genes, p53 and p16INK4A, SMZL is commonly characterized by the deletion of the long arm of chromosome 7 (7q21-33) and mutations of the NOTCH-2 pathway [12-14]. It has been postulated that there may be a tumor suppressor gene located within the deleted arm of chromosome 7 resulting in neoplastic activity [15]. The mutations associated with NOTCH receptors contribute to target gene expression, resulting in constitutive gene expression [16]. Once neoplastic activity through acquired mutations has occurred, the typical presentation of SMZL involves splenomegaly and lymphocytosis [17]. This patient’s presentation was atypical in that she did not show any of these signs [17]. SMZL normally expresses CD20, immunoglobulin M (IgM), and sometimes IgD, but this patient’s cytometry was notable for the expression of CD5, CD20, and CD23, which are seen in less than 20% of SMZLs [17]. In addition, hypocellular bone marrow, as seen in this patient, is only seen in approximately 10% of MDS cases [18].

In addition, it should be noted that about 10-15% of SMZL can undergo transformation to diffuse large B-cell lymphoma requiring further treatment and evaluation [19].

Whether this patient’s hydroxyurea use may have contributed to the resulting SMZL is difficult to presume, as research into its pathophysiology is limited. However, it would be reasonable to consider that it may have played a role in this patient’s hypocellularity as cell counts improved following cessation of hydroxyurea [20]. Drug-induced myelosuppression is plausible, as hydroxyurea is an anti-metabolite that hinders DNA synthesis by inhibiting ribonucleotide reductase, thus lending itself as a treatment for resistant leukemias, myeloproliferative disorders, including polycythemia vera and essential thrombocytosis [21]

This disruption in DNA synthesis can lead to genetic mutations, resulting in the potential of activating oncogenes and thus, malignancy; this has been a potential consequence with long-term use. The leukemogenic potential of hydroxyurea has been attributed to about 1-5% of cases while other studies state there is minimal, if any, association in the development of malignancy [21-23]. Thus, the side effect profile of hydroxyurea requires further studies; however, this case presentation offers support for its’ leukemogenic effect. It also offers support in hydroxyurea’s myelosuppressive nature, as the patient's cytopenia’s improved with the discontinuation of hydroxyurea.

Regardless of the pathophysiology that gives rise to SMZL, it has historically been treated with splenectomy in symptomatic patients and observation in asymptomatic patients [24]. Splenectomy is most efficacious in patients with localized symptoms and minimal dissemination [24]. Chemotherapy is typically reserved for relapsed and refractory cases. Recent improvements in therapy highlight rituximab, fludarabine, and pentostatin as effective monotherapies for B-cell lymphomas and rituximab, fludarabine, dexamethasone, and mitoxantrone as effective regimens for indolent lymphomas [25]. A 2018 phase II trial is evaluating the efficacy of rituximab and bendamustine as initial therapy [26].

It should be noted that there currently needs to be standardized treatment due to the paucity of prospective randomized trials. Retrospective studies have shown conflicting efficacy of varying therapies. A 2006 study of 70 patients endorsed rituximab use, with or without chemotherapy, due to its increased response rate, three-year survival, and failure-free survival, particularly in elderly patients with comorbidities and elevated CD20+ counts [27]. A 2012 study of 593 patients concluded that patient’s treated with splenectomy, rituximab, and chemotherapy, had worse outcomes than non-treated patients with no significant difference in survival rates [28]. A 2013 study of 85 patients endorsed rituximab usage over splenectomy due to better response rates, overall survival, and progression-free survival [29]. A 2018 study of 108 patients, endorsed rituximab as monotherapy with a 92% response rate and 10-year freedom from progression of >60% [9]. As such, treatment modalities vary based on patient presentation and rely on shared decision-making between oncology providers and patients.

## Conclusions

SMZL’s low incidence, indolent course, elusive diagnostic criteria, and limited evidence-based treatments represent a significant challenge for clinicians. In addition, research in secondary malignancy in the setting of hydroxyurea use is limited and not well- understood. This patient’s diagnosis of SMZL was particularly challenging given her atypical symptoms/presentation, resulting in a delay of diagnosis; Further exploration into atypical SMZL presentations and hydroxyurea’s potential in secondary malignancies is recommended such as further clinical research.
